# G Protein-Coupled Receptors in Irritable Bowel Syndrome: Mechanisms and Therapeutic Opportunities

**DOI:** 10.3390/ijms27020752

**Published:** 2026-01-12

**Authors:** Zhenya Zhu, Ziyu Liu, Yate He, Xiaorui He, Wei Zheng, Mizu Jiang

**Affiliations:** Department of Gastroenterology, Children’s Hospital, Zhejiang University School of Medicine, National Clinical Research Center for Children and Adolescents’ Health and Diseases, National Children’s Regional Medical Center, Hangzhou 310052, China

**Keywords:** irritable bowel syndrome, G protein-coupled receptors, immune regulation, microbiota–host crosstalk, metabolic signaling, pain processing

## Abstract

Irritable bowel syndrome (IBS) is a functional gastrointestinal disorder characterized by abdominal pain, altered motility, and visceral hypersensitivity. Emerging evidence implicates G protein-coupled receptors (GPCRs) as key integrators of microbial, immune, endocrine, and neural signals in IBS pathophysiology. This review summarizes recent advances in understanding how GPCRs mediate gut immune regulation, microbiota–host crosstalk, metabolic signaling, and pain processing in IBS. Recent studies show that microbial metabolites (e.g., short-chain fatty acids, biogenic amines, and lipid mediators) signal through GPCRs on immune cells, epithelia, and neurons to influence intestinal homeostasis. On immune cells and neurons, GPCRs also mediate signals from external substances (such as fats, sugars, histamine, etc.) to regulate immune and neural functions. And there are challenges and future directions in targeting GPCRs for IBS, including patient heterogeneity and the complexity of host–microbiome interactions. This review provides a mechanistic framework for GPCR-based therapies in IBS.

## 1. Introduction

Irritable bowel syndrome (IBS) affects about 5~10% of the population worldwide and is characterized by chronic abdominal pain and disturbed bowel habits in the absence of overt intestinal damage [1,2]. Its pathophysiology is multifactorial, involving dysregulated motility, visceral hypersensitivity, altered brain–gut signaling, low-grade immune activation, and microbial dysbiosis. The gastrointestinal tract is continuously exposed to a wide range of dietary nutrients, and accumulating evidence indicates that dietary modification can, at least in part, influence symptom severity in patients with IBS [3]. Among IBS patients, gut microbiota dysbiosis is an aspect that represents a key feature; moreover, the mutual influence between the immune and nervous systems also plays a significant role in the pathophysiology of IBS.

G protein-coupled receptors (GPCRs) constitute the largest family of cell-surface signaling proteins and are pivotal in relaying information from the gut lumen, microbiota, and host metabolism to intestinal cells [4]. Structurally, GPCRs are characterized by a conserved seven-transmembrane helical architecture with extracellular ligand-binding domains and intracellular loops and C-terminal tails [5]. Upon ligand engagement, GPCRs activate intracellular signaling cascades primarily through heterotrimeric G proteins and arrestins. GPCR-mediated signaling orchestrates myriad functions across organ systems, including sensory perception and neural transmission in the nervous system [6], metabolic regulation via hormonal receptors [4], immune cell chemotaxis [7] and activation via chemokine receptors [8].

GPCRs respond to a diverse spectrum of ligands—including neurotransmitters, hormones, dietary components, and microbiota-derived metabolites—and thereby shape immune responses, epithelial function, neuronal excitability, and metabolic regulation [9]. Recent work has revealed that gut microbes produce small molecules that act as ligands for host GPCRs, modulating gut–immune–brain interactions. For example, short-chain fatty acids (SCFAs) from microbiota bind to free fatty acid receptors (FFARs) on immune and epithelial cells [10,11], while indole derivatives and biogenic amines target serotonin and amine GPCRs [12]. Recent advances in GPCR biology, including insights into receptor trafficking and biased signaling, provide new opportunities to exploit GPCRs therapeutically in IBS [5,6,9].

This review synthesizes recent mechanistic and translational findings on GPCRs in four interlinked domains relevant to IBS: gut immune regulation, microbiota–host interactions, metabolic modulation, and visceral pain. We highlight promising therapeutic concepts and explicitly discuss current limitations and translational challenges.

## 2. GPCRs and Immune Regulation in the Gut

The gut immune system is a specialized mucosal network that balances tolerance to food and commensal microbes with defense against pathogens [13,14]. The epithelial layer, mucus, secretory IgA and antimicrobial peptides form a physical–chemical barrier that limits microbial contact and controls antigen uptake. Beneath the epithelium, innate immune cells—including macrophages, dendritic cells, neutrophils, and innate lymphoid cells—continuously sample luminal antigens and shape adaptive immune responses. In parallel, gut-associated lymphoid tissue (GALT), mesenteric lymph nodes, and tissue-resident T and B cells coordinate IgA production and regulatory T cell-dependent immune tolerance [15].

IBS has traditionally been classified as a functional gastrointestinal disorder, now also known as disorders of gut–brain Interaction (DGBI). However, accumulating evidence indicates that subtle immune dysregulation contributes to its pathogenesis. Mucosal biopsies from IBS patients reveal evidence of chronic low-grade inflammation: for example, increased numbers of lamina propria mast cells, T lymphocytes and other immune cells have been reported in IBS patients [16]. Notably, mast cells are often adjacent to enteric nerves and secrete mediators (histamine, serotonin, tryptase) that can sensitize sensory fibers, linking immune activation to abdominal pain and hypersensitivity [16,17]. Alterations in innate immune cell function have also been described: dendritic cells isolated from IBS mucosa exhibit enhanced release of tumor necrosis factor-α (TNF-α) and other proinflammatory cytokines [18], while intestinal macrophages display an activated phenotype that may influence barrier integrity and cytokine signaling [16,18]. Consistent with these observations, elevated levels of proinflammatory cytokines—including TNF-α, IL-1β, and IL-6—have been detected in both mucosal tissues and circulation of IBS patients compared with healthy controls [19], supporting the concept of a persistent low-grade inflammatory milieu.

GPCRs expressed on intestinal immune cells are key regulators of mucosal immunity and inflammation. For example, the SCFA receptor FFAR2 (also known as GPR43) is critical for gut immune homeostasis. Chun et al. demonstrated that mice lacking *Ffar2* exhibit markedly altered populations of group 3 innate lymphoid cells (ILC3s) in the colon, accompanied by impaired mucosal immune defense [10]. SCFAs produced by fiber-fermenting bacteria thus act through FFAR2 to maintain IL-22–producing ILC3s, suggesting dietary modulation of this pathway could bolster gut defenses. Altered SCFA profiles have been reported in IBS patients [20], although findings remain inconsistent across studies, likely reflecting clinical and microbial heterogeneity within IBS populations [21].

Similarly, the glucagon-like peptide-1 receptor (GLP-1R) on gut intraepithelial lymphocytes (IELs) exerts dual functions in metabolic and immune regulation [22]. Wong et al. reported that activation of GLP-1R on IELs regulates systemic metabolic homeostasis and glucose balance, while also shaping gut microbial composition and restraining T cell-mediated inflammation [22,23]. These findings highlight a dual role for GLP-1R in linking metabolism and immunity at the mucosal interface. In addition, activation of GLP-1R in central neurons has been shown to suppress Toll-like receptor–induced plasma TNF-α production [24], further highlighting the importance of brain–gut–immune interactions in regulating intestinal homeostasis.

Several GPCRs on innate immune cells respond directly to microbiota-derived metabolites to limit inflammation. The niacin/SCFA receptor GPR109A, expressed on colonic macrophages, mediates anti-inflammatory effects: dysbiosis reduces butyrate levels and thus GPR109A signaling, which can exacerbate inflammation [25]. Correspondingly, activating GPR109A by restoring its ligands is proposed as a therapeutic strategy in inflammatory bowel disease (IBD) and potentially IBS subtypes associated with immune activation. Likewise, Li et al. demonstrated that butyrate inhibits neutrophil overactivation, reducing mucosal inflammation in an experimental IBD model [26]. Although this work focused on IBD, the underlying mechanism—microbiota-derived SCFAs acting through GPCR-mediated pathways—may also be pertinent to IBS, where subtle neutrophil dysregulation has been reported.

GPCRs additionally regulate dendritic cell (DC) function in the intestinal mucosa. Oguro-Igashira et al. showed that pyruvate, a common metabolic intermediate, binds to GPR31 on intestinal DCs and promotes the extension of transepithelial dendrites [27]. This enhances antigen sampling from the gut lumen and bolsters immune surveillance. In an IBS context, impaired antigen processing or aberrant immune activation could conceivably arise from altered GPCR signaling in DCs.

The table below summarizes selected GPCRs on immune and epithelial cells, their known ligands, cell types, and roles in gut immunity or inflammation. These examples illustrate how microbial and host metabolites engage GPCRs to tune mucosal immune balance and barrier function (Table 1). Collectively, these examples illustrate how host- and microbiota-derived metabolites engage GPCRs to fine-tune mucosal immune balance and barrier function.

These studies collectively underscore that GPCRs on immune cells sense both host and microbial signals to restrain inflammation and shape immunity. In IBS, where mucosal immune alterations can contribute to symptom generation, targeting such GPCR pathways (for example, enhancing FFAR2 signaling with SCFAs or modulating GLP-1R) may restore immune homeostasis. Nevertheless, significant challenges remain, including the need to define cell-specific effects and to translate findings derived largely from IBD models to the low-grade inflammatory context characteristic of IBS.

## 3. GPCRs as Mediators of Microbiota–Host Interactions

The gut microbiota constitutes a complex metabolic organ that profoundly influences host physiology. Gut bacteria digest dietary fibers and other nutrients that the host cannot, generating metabolites that serve as energy sources for colonocytes and potent signaling molecules [30,31]. Microbial products also “educate” the immune system and help maintain intestinal barrier integrity, while dysbiosis—alterations in microbiota composition or function—is linked to low-grade inflammation and loss of tolerance. Indeed, patients with IBS consistently show altered microbiota diversity and metabolite profiles compared to healthy individuals, supporting a role for dysbiosis in IBS pathogenesis [32]. Beyond local effects, gut microbes also influence the gut–brain axis by producing neuroactive compounds, such as γ-aminobutyric acid (GABA), serotonin, and dopamine, and by modulating vagal and immune signaling to the central nervous system [31,33].

A key mechanism by which gut microbes communicate with the host is through the production of small-molecule metabolites that activate host GPCRs. A striking example is tryptamine, a microbial metabolite derived from tryptophan. Bhattarai et al. showed that tryptamine activates an epithelial Gq-coupled receptor (identified as the serotonin receptor 5-HT_4_R) in colonocytes, leading to increased Cl^−^ and fluid secretion [12]. This provides a direct mechanism by which gut bacteria influence gut motility and secretion through GPCR signaling. Similarly, microbial-derived kynurenic acid has been shown to activate GPR35, a class A GPCR expressed on intestinal epithelial cells and lamina propria macrophages, thereby promoting mucosal repair and limiting inflammatory responses [34].

Certain gut bacteria can generate even more complex GPCR ligands. Chang et al. reported that human gut *Clostridia* can conjugate neurotransmitters (dopamine or serotonin) with dietary or host fatty acids, producing hybrid molecules that agonize various GPCRs like EB12 (GPR183) [35]. These “molecular mimics” exemplify the complexity of microbe-host crosstalk: bacterial enzymes create novel ligands that may modulate host immune, metabolic, or neuromotor pathways via GPCRs.

Microbial metabolites may also hijack host GPCRs in pathogenic ways. *Enterococcus*-derived tyramine, for instance, binds to the α2A-adrenergic receptor on intestinal stem cells, disrupting their regenerative function and worsening colonic inflammation [28]. Similarly, phenylacetylglutamine (PAGln), a metabolite from gut microbes, was recently identified as an allosteric modulator of the β2-adrenergic receptor [36]. Although this study primarily focused on cardiovascular outcomes, it underscores the broader concept that bacterially produced metabolites can modulate canonical GPCR signaling pathways with systemic consequences.

These microbial–GPCR interactions often impact intestinal barrier and immune homeostasis. For example, butyrate (a classic SCFA ligand for FFAR3/GPR41) was shown to protect colonocytes from adherent-invasive *E. coli*—induced mitochondrial damage via FFAR3 signaling [11]. Restoration of such GPCR-mediated protection by butyrate suggests therapeutic angles in dysbiosis. Moreover, GPCRs such as GPR109A, which responds to microbiota-derived niacin and butyrate, play a key role in orchestrating macrophage-mediated inflammatory responses and tolerance in the gut [25].

Selected examples of microbial metabolites and their corresponding GPCR targets in the intestine are summarized in Table 2. These interactions emphasize how shifts in microbiome composition or metabolite levels could tip the balance toward dysregulation in IBS. These examples illustrate that microbial communities shape host signaling through GPCR ligands, creating a complex metabolite–GPCR network. Perturbations of this network could underlie IBS symptoms. Accordingly, targeting the microbiota–GPCR interface, for example, through dietary interventions that modify SCFA profiles or through pharmacological modulation of specific GPCRs, represents a promising avenue for future therapeutic development.

## 4. GPCR Signaling and Metabolic Modulation

GPCRs not only regulate immunity but also coordinate metabolic cues in the gut. Broader metabolic disturbances have been implicated in IBS. Dietary factors are major modulators of IBS symptoms and pathophysiology; structured interventions—most notably the low fermentable oligosaccharides, disaccharides, monosaccharides and polyols (FODMAP) diet—reduce global symptoms, bloating and abdominal pain in many patients, likely by decreasing luminal fermentation, modifying microbiota-derived metabolites and downstream GPCR-mediated signaling [39,40]. Dietitian-led reintroduction and personalization are recommended to preserve nutritional adequacy and long-term efficacy [3]. Consistent with these clinical observations, metabolomics studies have revealed widespread alterations in metabolic profiles in IBS patients, despite only modest changes in overall gut microbiota composition [41]. A recent multi-omics analysis identified hundreds of differential serum metabolites in IBS and found dysregulated tryptophan–serotonin metabolism correlating with symptom severity [41].

Enteroendocrine cells express a broad array of nutrient-sensing GPCRs that detect luminal carbohydrates, lipids, and peptides, thereby coupling nutrient availability to hormonal and neural outputs that regulate digestion, absorption, and systemic metabolism [42]. For example, enterochromaffin cells express GPCRs for hormones and metabolites. Lund et al. found that GLP-1 and SCFA significantly influence serotonin production by these cells [43]. Through this pathway, gut peptides and microbiota-derived SCFAs modulate gut motility and appetite regulation, highlighting a nexus between microbial signals, enteroendocrine responses, and host metabolic homeostasis.

Another intriguing link is GPR17 in the intestine. Yan et al. reported that loss of *Gpr17* enhances GLP-1 secretion from intestinal L-cells, improving glucose tolerance [44]. Since GLP-1 slows gastric emptying and affects motility, GPR17 may indirectly influence IBS symptoms, especially in patients with coexistent metabolic syndrome. Conversely, GLP-1 analogs have been tested for IBS: clinical studies suggest GLP-1 can dampen migrating motor complexes and slow transit in IBS patients [45,46]. Moreover, adherence to a low-FODMAP diet has been associated with increased circulating GLP-1 levels in IBS [47]. The GLP-1R agonist ROSE-010 can reduce pain in IBS patients at different doses, but it may also cause side effects such as nausea, vomiting, and headaches [48,49]. Together, these findings underscore the relevance of GPCRs that regulate incretin signaling to both metabolic control and gastrointestinal function in IBS.

Bile acids (BAs) are another important factor in IBS metabolic pathways. Malabsorption of BAs is found in roughly 25% of patients with diarrhea-predominant IBS (IBS-D) [50], leading to excess luminal BAs that promote secretory diarrhea. Under physiological conditions, most primary bile acids are reabsorbed in the ileum, while those reaching the colon are converted by gut microbes into secondary bile acids with distinct effects on mucosal secretion and motility [50]. Disruption of this BA–microbiota crosstalk can exacerbate symptoms—for instance, IBS-D patients often harbor an overabundance of bile-deconjugating *Clostridia* (e.g., *Clostridium scindens*), which is linked to elevated fecal BAs and diarrhea [51,52]. Diet-derived GPCR ligands also modulate gut tissue structure. Secondary bile acids, produced by microbiota from primary bile acids, activate nuclear and membrane receptors to regulate epithelial turnover.

Overall, GPCR signaling integrates nutritional and microbial cues to adjust gut physiology. Dysregulation of these pathways could contribute to IBS symptoms like postprandial discomfort or altered transit. For instance, aberrant nutrient sensing or enteroendocrine responses may underlie symptom flare-ups. From a therapeutic perspective, these insights suggest that GPCR-targeted strategies—such as GLP-1 receptor agonists or bile acid-modulating agents—may hold potential for IBS management, although careful patient selection and safety considerations are required. Moreover, the nutrient–GPCR axis highlights the value of dietary modification: increasing fiber to boost SCFAs may engage FFARs to improve gut motility and immunity. Future research should further define which metabolic GPCRs are most perturbed in IBS and how they can be harnessed for therapy [53].

## 5. GPCRs and Visceral Pain Pathways

A hallmark feature of IBS is visceral hypersensitivity, whereby patients exhibit exaggerated pain responses to physiological or innocuous gastrointestinal stimuli. Visceral hypersensitivity involves complex interactions between peripheral and central nervous systems, as well as the gut–brain axis. Peripheral mechanisms contributing to visceral hypersensitivity include inflammation, altered gut microbiota, and immune activation. Studies have shown that stress can activate the sympathetic nervous system while inhibiting the vagus nerve, leading to increased intestinal permeability and inflammation, which in turn heighten visceral sensitivity [53]. Additionally, mast cell dysfunction in the gut can disrupt epithelial barrier function, further contributing to pain perception [54].

Central mechanisms involve changes in brain regions involved in pain processing. Functional imaging studies have demonstrated increased activity in areas such as the anterior cingulate cortex and the insula in IBS patients, suggesting alterations in central pain processing [55]. The gut–brain axis serves as a bidirectional communication pathway between the gastrointestinal tract and the central nervous system. Disruptions in this communication, due to factors like stress, inflammation, or microbial dysbiosis, can exacerbate visceral hypersensitivity and contribute to IBS symptoms [55].

GPCRs expressed in sensory neurons and gut neuroimmune circuits are central to pain modulation. Bacterial infection or toxins can elicit intestinal, antigen-specific IgE that sensitizes tissue mast cells. Subsequent ingestion of the cognate food antigen provokes mast-cell degranulation and H1-receptor-dependent visceral afferent sensitization, producing meal-induced abdominal pain in mice and clinically observed local mucosal reactions in IBS patients during feeding [56]. Similarly, gut bacteria-derived histamine can engage the histamine H_4_ receptor (H_4_R) on mast cells and sensory neurons, triggering mast cell accumulation and visceral hyperalgesia via a mast cell–nerve pathway [57].

The Mas-related G protein-coupled receptor (MRGPR) family is primarily expressed in sensory neurons and immune cells in the human body, playing a role in the perception of itching, pain, and inflammation [58]. Eight subtypes are expressed in humans, including MRGPRD, MRGPRE, MRGPRF, MRGPRG, and MRGPRX1-X4. Batazova et al. demonstrated that 5-oxo-eicosatetraenoic acid (5-oxoETE), a lipid mediator, activates MRGPRD on nociceptive neurons, triggering pain responses in constipation-predominant IBS (IBS-C) [37]. This identifies a novel pain pathway: a polyunsaturated fatty acid metabolite binds MRGPRD to enhance visceral nociception, suggesting that blocking MRGPRD could alleviate pain in IBS-C.

Another GPCR implicated in IBS pain is Mas-related G protein-coupled receptor X2 (MRGPRX2), which is expressed on intestinal mast cells. In colonic tissue from IBS patients, MRGPRX2 expression is increased more than tenfold compared with controls [59]. Decraecker et al. reported that MRGPRX2-mediated mast cell activation is enhanced in IBS patients, despite unchanged mast cell numbers [38]. Activation of MRGPRX2 by neuropeptides such as substance P leads to mast cell degranulation and sensitization of transient receptor potential vanilloid 1 (TRPV1) channels on neurons. The upregulation of this pseudo-allergic pathway in IBS supports the idea that MRGPRX2 is a contributor to abdominal pain and hypersensitivity. The antagonist of MRGPRX2, EP262, is undergoing clinical trials for the treatment of chronic inducible urticaria, chronic spontaneous urticaria, and atopic dermatitis [60,61]. Blocking MRGPRX2 or its agonists could therefore represent a novel analgesic strategy in IBS.

Mast cells thus emerge as central effectors linking luminal antigens and microbial mediators to visceral hypersensitivity through IgE-dependent degranulation and GPCR-mediated sensing pathways, including H1/H4 receptors and MRGPRX2. The release of histamine, tryptase, and prostaglandins sensitizes afferent neurons and amplifies pain signaling [54]. Future therapeutic directions include mast-cell-selective GPCR antagonists or stabilizers, microbiome- and diet-based modulation of mast-cell activation, and patient stratification by mucosal biomarkers and single-cell profiling to enable precision therapies.

GPCR-mediated modulation of sensory neuropeptides further contributes to IBS pain. Calcitonin gene-related peptide (CGRP) is a key nociceptive neuropeptide. Pujo et al. demonstrated that the gut microbiota influences visceral sensitivity by regulating CGRP production [62]. Although CGRP acts through its own GPCR (the CGRP receptor), this finding underscores a microbe–neural axis in IBS pain. Therapeutically, monoclonal antibodies against CGRP are effective in migraine and are being explored for gastrointestinal pain. GPR35 agonists reduce pain responses triggered by transient receptor potential A1 (TRPA1) channels, particularly in the colon. By suppressing substance P release, GPR35 agonists effectively reduce neuroinflammation and nociceptive signals, highlighting their potential as a treatment for colonic pain [63].

More generally, GPCRs have long been recognized as analgesic targets. Gottesman-Katz et al. reviewed that GPCRs involved in gastrointestinal pain pathways—including opioid, cannabinoid, serotonin, and adrenergic receptors—are candidate targets for new therapies [64]. For example, peripheral κ-opioid agonists (GPCR ligands) are under trial to relieve IBS pain without central side effects. Adenosine receptors also modulate pain: adenosine A1 or A2 receptor agonists can have analgesic effects in visceral models. Shakya et al. note the role of adenosine receptors in IBS and suggest that targeting the adenosine pathway may benefit IBS symptoms [29]. In addition, the GLP-1 analog liraglutide markedly attenuated experimentally induced visceral hypersensitivity in rat models [65], further supporting the concept that peripheral GPCR modulation may offer effective and safer pain control in IBS.

These examples demonstrate that GPCR signaling is tightly linked to visceral nociception. Novel findings (e.g., MRGPRD and MRGPRX2 pathways) open opportunities for targeted pain relief in IBS. However, receptor specificity and off-target effects are crucial considerations in developing clinically useful analgesics.

## 6. Conclusions and Future Directions

**Figure 1:**
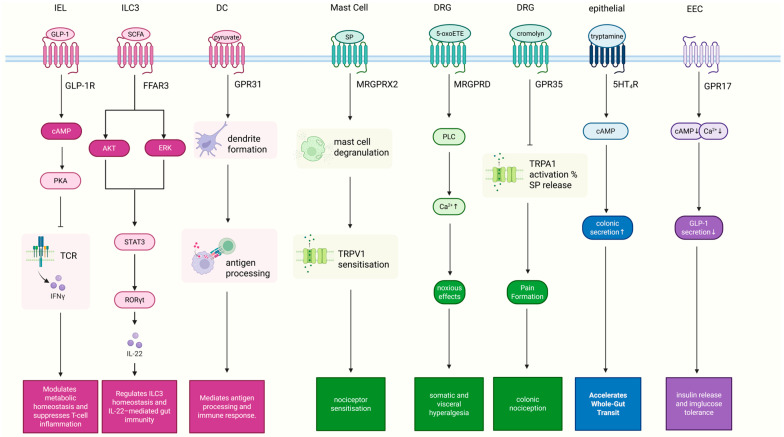
Schematic overview of G protein-coupled receptor (GPCR)-mediated signaling pathways relevant to IBS, highlighting immune regulation, epithelial function, and visceral nociception. GLP-1—glucagon-like peptide-1; GLP-1R—GLP-1 receptor; FFAR3—free fatty acid receptor 3; MRGPR—Mas-related G protein-coupled receptor; SP—substance P; 5-HT_4_R—serotonin receptor 4; EEC—enteroendocrine cell; IEL—intraepithelial lymphocyte; DRG—dorsal root ganglion; cAMP—cyclic adenosine monophosphate; PKA—protein kinase A; PLC—phospholipase C; TCR—T cell receptor; IFNγ—interferon-gamma; STAT3—signal transducer and activator of transcription 3; RORγt—retinoid-related orphan receptor-γt; IL-22—interleukin-22; ↑ upregulated; ↓ downregulated. (Created in BioRender. Zhu, Z. (2026) https://BioRender.com/ahd1s1o).

Recent advances have delineated multiple GPCR-mediated mechanisms that link microbial metabolism, immune regulation, enteroendocrine signaling, and sensory pathways in IBS (Figure 1). These insights create a mechanistic framework for novel therapies that target GPCRs directly or modulate their endogenous ligands via diet or microbiota interventions. Accumulating evidence positions GPCRs as central integrators of immune, microbial, metabolic, and neuro-sensory signaling in IBS [4,9,64]. Through sensing microbiota-derived metabolites, dietary components, and host mediators, GPCRs expressed on epithelial cells, immune cells, enteroendocrine cells, and sensory neurons coordinate mucosal immunity, barrier integrity, metabolic regulation, and visceral pain perception [10,11,21,25,26].

IBS comprises multiple clinical subtypes, including IBS-C, IBS-D, mixed bowel habits IBS (IBS-M), and unclassified IBS (IBS-U), which are likely characterized by distinct underlying mechanisms [1]. A GPCR-targeted therapy may improve symptoms in one subtype while worsening another (e.g., a prokinetic 5-HT_4_R agonist may help IBS-C but exacerbate IBS-D) [66,67]. Therefore, subtype-specific trial designs and stratified analyses are essential. And IBS is chronic; long-term benefit–risk data for many GPCR-targeted approaches are lacking [2]. Historical examples (e.g., restrictions on certain serotonergic agents) underscore the need for careful safety monitoring in extended trials.

Inter-individual and temporal variability in the microbiome influences the production of GPCR ligands (SCFAs, histamine, bile acid metabolites), which in turn affects therapy responsiveness. Integrating baseline microbiome and metabolomic profiling into trials may improve patient selection and reproducibility.

The GPCR superfamily is large and functionally overlapping [68]; many receptors couple to multiple signaling pathways and have tissue-specific roles. This complexity raises the risk of off-target effects and complicates candidate selection. Approaches such as biased agonism and allosteric modulation may increase selectivity and reduce adverse effects.

While preclinical data are compelling, several translational hurdles must be acknowledged. Much mechanistic evidence arises from rodent models, in vitro systems, or organoids that do not fully recapitulate human IBS, particularly its psychological comorbidities and chronic, low-grade inflammatory milieu. Consequently, preclinical efficacy may not predict clinical benefit, and more well-designed human mechanistic and early-phase trials are required. To advance precision medicine, clinical studies should incorporate biomarkers (microbial metabolites, receptor expression patterns, inflammatory markers) to stratify patients and monitor target engagement [68]. Multi-omic and single-cell profiling approaches can help define endotypes amenable to specific GPCR interventions.

While GPCR-based therapeutics are developing, current evidence supports several practical interventions that modulate GPCR-relevant pathways and may be recommended in clinical practice [3]: Dietary modification (e.g., low-FODMAP approaches) can reduce fermentable substrate availability, altering microbial metabolite profiles and improving symptoms in many patients; dietitian-guided reintroduction is advised to preserve nutrition and long-term adherence [3,39,40]. Microbiome-modulating therapies (selected probiotics, prebiotics, or engineered strains) may shift metabolite production (e.g., increase SCFA output) to engage beneficial GPCR signaling, although efficacy varies and should be individualized. Psychological and behavioral therapies (cognitive-behavioral therapy, gut-directed hypnotherapy) address central drivers and brain–gut interactions that interact with peripheral signaling pathways. These lifestyle and behavioral strategies complement receptor-targeted treatments and can be integrated into a personalized care plan.

To accelerate translation, we recommend: (1) well-powered, mechanistically informed clinical trials that incorporate biomarker and microbiome profiling; (2) development of selective biased agonists and allosteric modulators to improve receptor specificity; (3) longitudinal studies to evaluate long-term efficacy and safety; and (4) combinatorial approaches that pair receptor modulators with diet or microbiome interventions tailored to patient endotypes. In summary, GPCRs provide convergent nodes linking microbial, metabolic, immune, and neural systems in IBS. With rigorous clinical evaluation and precision-guided strategies, GPCR-based approaches have the potential to move IBS care beyond symptom control toward mechanism-directed, individualized therapy.

## Figures and Tables

**Table 1:** GPCRs associated with gut immunity.

GPCR (Alias)	Ligand(s)	Cell Type(s)	Role(s) in IBS/Gut Immunity	References
FFAR2 (GPR43)	SCFAs (acetate, propionate)	Colonic ILC3	Regulates ILC3 homeostasis and IL-22-mediated gut immunity	[10]
GLP-1R	GLP-1 (gut hormone)	Intraepithelial T cells	Modulates metabolic homeostasis and suppresses T cell-mediated inflammation	[22]
GPR109A (HCAR2)	Butyrate (microbial SCFA)	Macrophages (lamina propria)	Anti-inflammatory signaling; maintains barrier integrity	[25]
GPR31	Pyruvate (metabolite)	Intestinal dendritic cells	Promotes transepithelial dendrite formation and antigen sampling	[27]
α_2A_-Adrenergic receptor	Tyramine (Enterococcus metabolite)	Intestinal stem cells	Tyramine–GPCR interaction impairs stem cell regeneration, exacerbating colitis	[28]
Adenosine A2A/A2B receptor	Adenosine (endogenous)	Immune cells (mast cell, macrophages)	Modulates gut inflammation and visceral pain; implicated in IBS	[29]

**Table 2:** GPCRs associated with IBS–Host crosstalk.

GPCR (Alias)	Ligand(s)	Cell Type(s)/Location	Role(s) in IBS/Host Physiology	References
5-HT_4_ receptor	Tryptamine (from *Ruminococcus*)	Colonic epithelial cells	Increases colonic fluid secretion; links microbiota to motility	[12]
FFAR3 (GPR41)	Butyrate, propionate	Colonic epithelium	Preserves epithelial mitochondria and barrier function	[11]
MRGPRD	5-oxoETE (arachidonic acid metabolite)	Visceral sensory neurons	Mediates nociceptive signaling in IBS-C; drives constipation-predominant pain	[37]
MRGPRX2	Substance P, endogenous peptides	Intestinal mast cells	Pseudo-allergic mast cell activation; contributes to visceral pain	[38]
α_2A_-Adrenergic receptor	Tyramine (*Enterococcus*)	Intestinal stem cells	Impairs epithelial regeneration; exacerbates inflammation	[28]
β_2_-Adrenergic receptor	Phenylacetylglutamine (microbial)	Various (heart, gut)	Allosteric modulation affects adrenergic signaling (stress response)	[36]
Adenosine A_2A_/A_2B_ receptor	Adenosine (microbial and host)	Gut immune cells, neurons	Regulates motility, pain, and inflammation; implicated in IBS pathogenesis	[29]

## Data Availability

No new data were created or analyzed in this study. Data sharing is not applicable to this article.
